# Kinematic assessment of an elastic‐core cervical disc prosthesis in one and two‐level constructs

**DOI:** 10.1002/jsp2.1040

**Published:** 2018-12-17

**Authors:** Richard D. Guyer, Leonard I. Voronov, Robert M. Havey, Saeed Khayatzadeh, Gerard Carandang, Kenneth R. Blank, Stephanie Werner, Josh Rubin, Nick Padovani, Avinash G. Patwardhan

**Affiliations:** ^1^ Texas Back Institute Plano Texas; ^2^ Loyola University Chicago Maywood Illinois; ^3^ Edward Hines Jr. VA Hospital Hines Illinois; ^4^ K2M Inc. Leesburg Virginia

**Keywords:** biomaterials, biomechanics, degeneration, disc arthroplasty, kinematics, motion preservation

## Abstract

**Introduction:**

Anterior cervical discectomy and fusion has been associated with the development of adjacent segment degeneration (ASD), with clinical incidence of approximately 3% per year. Cervical total disc arthroplasty (TDA) has been proposed as an alternative to prevent ASD.

**Hypotheses:**

TDA in optimal placement using an elastic‐core cervical disc (RHINE, K2M Inc., Leesburg, Virginia) will replicate natural kinematics and will improve with optimal vs anterior placement.

**Methods:**

Seven C3‐T1 cervical cadaver spines were tested intact first, then after one‐level TDA at C5‐C6 anterior placement, after TDA at C5‐C6 optimal placement, after two‐level TDA at C5‐C6 and C6‐C7 optimal placement, and finally after two‐level TDA at C5‐C6 lateral placement and C6‐C7 optimal placement. The specimens were subjected to: Flexion‐Extension moments (+1.5 Nm) with compressive preloads of 0 N and 150 N, lateral bending (LB) and axial rotation (AR) (+1.5 Nm) without preload.

**Results:**

*C5‐C6 TDA in* optimal placement resulted in a non‐significant increase in flexion‐extension ROM compared to intact under 0 N and 150 N preload (*P* > 0.05). Both LB and AR ROM decreased with arthroplasty (*P* < 0.01). *O*ptimal placement of *C6‐C7 TDA* resulted in an increase in flexion‐extension ROM with preload compared to intact (*P* < 0.05) while LB and AR ROM decreased with arthroplasty (*P* < 0.01).

**Conclusion:**

This six degree of freedom elastic‐core disc arthroplasty effectively restored flexion‐extension motion to intact levels. In LB the TDA maintained 42% ROM at C5‐C6 and 60% at C6‐C7. In AR 57% of the ROM was maintained at C5‐C6 and 70% at C6‐C7. These findings are supported by literature which shows cervical TDA results in restoration of approximately 50% ROM in LB and AR, which is a multifactorial phenomenon encompassing TDA design parameters and anatomical constraints. Anterior placement of this viscoelastic TDA device shows motion restoration similar to optimal placement suggesting its design may be less sensitive to suboptimal placement.

## 1 INTRODUCTION

Anterior cervical discectomy and fusion (ACDF) is considered to be the “gold‐standard” surgical procedure for the treatment of symptoms caused by cervical spondylosis and disc herniation. Degeneration of spinal segments adjacent to a previous fusion, termed adjacent segment disease (ASD), has been attributed in part to the initial fusion. The clinical incidence of symptomatic adjacent segment degeneration is estimated to be about 2.9% per year for the first 10 years after fusion. Approximately two‐thirds of these patients require re‐operation.^1^ Cervical total disc arthroplasty (TDA) has been proposed as an alternative to fusion to prevent adjacent segment degeneration and is now challenging ACDF as the “gold‐standard”.

Biomechanical studies have demonstrated that TDA can replicate physiologic motion at the index level and allow normal kinematics at adjacent levels.^2^, ^3^, ^4^ Recent prospective, randomized studies using validated outcome measures including neurologic success, pain, function, and return to work have shown that treatment of single‐ and two‐level radiculopathy or myelopathy with cervical TDA results in outcomes superior to ACDF.^5^, ^6^, ^7^, ^8^, ^9^, ^10^

This biomechanical study sought to characterize the kinematics of human cervical spine specimens implanted with TDA at the C5‐C6 and C6‐C7 levels. We tested the hypotheses that (a) cervical disc replacement using an elastic‐core cervical disc prosthesis (RHINE, K2M Inc., Leesburg, Virginia) will replicate the normal intact kinematics of the cervical spine, and (b) range of motion of the implanted segment will be maximized with optimal implant placement in the sagittal and coronal planes.

## 2 METHODS

### 2.1 Specimens and experimental set‐up

Seven human cervical (C3‐T1) cadaveric spine specimens (age: 41.0 ± 10.2 years) were tested **(**Table 1 **)**. The specimens had no radiographic signs of metastatic disease or bridging osteophytes. The paravertebral muscles were dissected and intervertebral discs, ligaments and bony structures were left intact. The specimens were wrapped in saline soaked towels to prevent dehydration of the soft tissues and testing was performed at room temperature.

**Table 1:** Specimen demographics

Specimen #	Age (years)	Sex	C5‐C6 TDA height (mm)	C6‐C7 TDA height (mm)
1	29	M	6	6	
2	31	M	6	7	
3	58	F	6	6	
4	47	M	6	6	
5	40	M	6	6	
6	46	M	6	6	
7	36	F	6	6	
**Mean**	**41.0**	**5 M, 2 F**			
SD	10.2				

The C3 and T1 vertebrae were anchored in cups using bone cement and pins. Specimens were fixed to the test apparatus at the caudal end (T1) and were free to move in any plane at the proximal end (C3). Moment loading was achieved by controlling the flow of water into and out of bags attached to loading arms fixed to the C3 vertebra.^11^ Flexion‐extension and lateral bending utilized a variable force at a distance to apply the moments necessary to produce motion. This technique assures that the testing apparatus does not constrain the motion of the specimen or contribute motion artifact. Axial rotation was performed using a pure axial rotation moment. Due to the low mass of the apparatus attached to the specimen (0.12 kg), no counter balance was necessary. The apparatus allowed continuous cycling of the specimen between specified maximum moment endpoints in flexion‐extension, lateral bending, and axial rotation.

**Figure 1:**
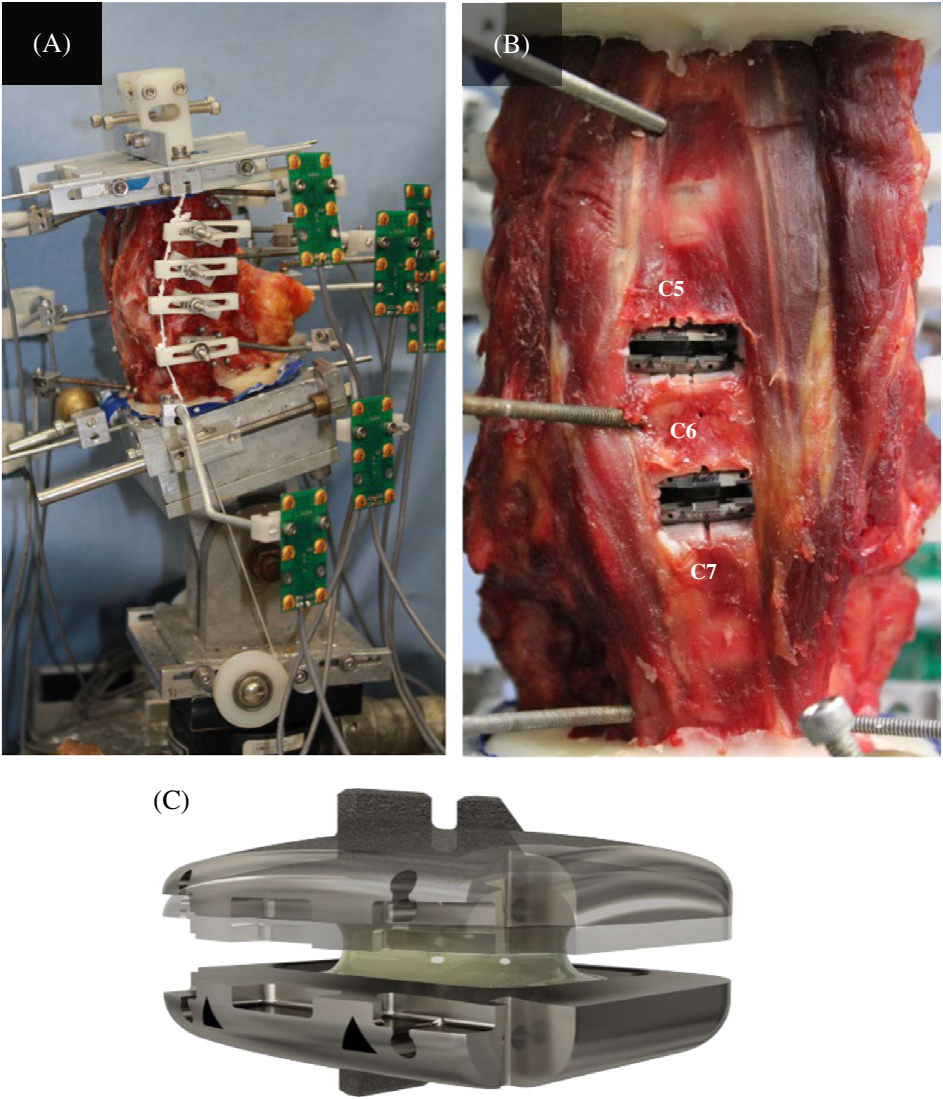
(A) Experimental setup showing C3‐T1 specimen with follower load cable and cable guides visible. (B) Surgical implantation of RHINE TDA at C5‐C6 and C6‐C7. Note the lateral placement of the TDA at C5‐C6. (C) Rhine TDA showing flexible polymer core and keels for bone fixation

The motion of the C3 to T1 vertebrae was measured using an optoelectronic motion measurement system (Optotrak Certus, Northern Digital, Waterloo, Ontario). In addition, bi‐axial angle sensors (Model 902‐45, Applied Geomechanics, Santa Cruz, CA) were mounted on each vertebra to allow real‐time feedback for the optimization of the preload path. A six‐component load cell (Model MC3A‐6‐250, AMTI Inc., Newton, Massachusetts) was placed under the specimen to measure the applied compressive preload and moments (Figure 1A). Fluoroscopic imaging (GE OEC 9800 Plus digital fluoroscopy machine) was used to measure intact disc heights prior to implantation and in flexion and extension to monitor vertebra and implant motion.

The follower load technique was used to apply compressive preload to the cervical spine during the range of motion experiments in flexion and extension.^11^, ^12^, ^13^, ^14^, ^15^, ^16^ The compressive preload was applied along the path that follows the lordotic curve of the cervical spine. This allowed the cervical spine to support physiologic compressive preloads without damage or instability.

The follower load was applied using bilateral loading cables that were attached to the cup holding the C3 vertebra. The cables passed freely through guides anchored to each vertebra and were connected to a loading apparatus under the specimen. The cable guides allowed anterior‐posterior adjustments of the follower load path within a range of approximately 10 mm. The preload path was optimized by adjusting the cable guides to minimize changes in cervical lordosis when a compressive load of 150 N was applied to the specimen in a moderately flexed posture. The preload path was considered optimized when the preload application from 0 N to 150 N produced no more than 0.3° of segmental motion and 0.3° of motion form C3 to T1.^11^, ^13^, ^14^

### 2.2 Experimental protocol

**Figure 2:**
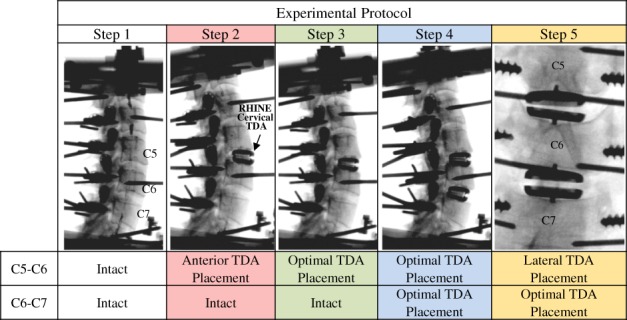
Experimental protocol: Step 1) intact, Step 2) anterior TDA placement at C5‐C6, Step 3) optimal TDA placement at C5‐C6, Step 4) optimal TDA placement at C6‐C7, Step 5) lateral TDA placement at C5‐C6

The specimens were tested under the following conditions (Figure 2); i) intact, ii) anterior placement of the TDA at C5‐C6, iii) optimal placement of the TDA at C5‐C6, iv) optimal placement of TD*A* at C6‐C7, v) lateral placement of TDA at C5‐C6.

The TDA tested in this work was an elastic‐core cervical disc prosthesis (RHINE, K2M Inc.). This TDA is a one‐piece non‐ articulating prosthesis with titanium alloy endplates which have a plasma sprayed titanium coating for integration with the bony endplates. The core is an elastomeric polyurethane sized and intended to mimic the mechanical properties (stiffness and ultimate strength) of an intact cervical disc.

Anterior placement was achieved by placing the prosthesis midline 2 mm anterior to the midline of the intervertebral disc space as determined radiographically on lateral fluoroscopy. Optimal placement was achieved by tapping the TDA into the disc space until the center of the prosthesis was aligned with the middle of the intervertebral disc space in the sagittal plane using fluoroscopy. Lateral placement at C5‐C6 TDA was achieved by removing the TDA, followed by reinsertion approximately 2 mm laterally from the midline of the intervertebral disc space and confirmed radiographically on antero‐posterior view (Figure 1B).

In each condition, the specimens were subjected to the following loads: Flexion‐Extension (FE) moments (±1.5 Nm) with compressive preloads of 0 N and 150 N, and lateral bending (LB) and axial rotation (AR) (±1.5 Nm) with compressive preloads of 0 N. These moment values (±1.5 Nm) are within the range of moments used in previous biomechanical studies of human cervical spine segments.^11^, ^12^, ^13^, ^14^ Loading was performed at a quasi static rate (0.2 Nm/second) to allow sufficient viscoelastic relaxation.

First, the baseline range of motion of the intact specimen was determined in FE, LR, and AR under 0 N external preload. The load‐displacement data was collected until two reproducible load‐displacement loops were obtained. This required a maximum of three loading cycles. Following optimization of the preload path, a range of motion test on the intact spine was repeated in FE for 150 N compressive preload.

After testing the intact spine, a discectomy was performed using standard instruments. The endplates was preserved but cleaned of all cartilage. The TDA was implanted at the C5‐C6 level using instrumentation and technique provided by the manufacturer. Trial sizes were used to estimate the size of the disc space for correct TDA height selection. Proper placement of the device in each protocol step was confirmed by fluoroscopy. The specimens were tested in FE, LB, and AR as described above after each protocol step.

### 2.3 Data analysis

The motion data was analyzed in terms of range of motion (ROM) at the implanted and adjacent segments for different simulated conditions. The load vs displacement curves were analyzed to determine the quantity of angular motion. In addition, quality of motion was assessed through measure of the neutral zone and segmental high flexibility zone (HFZ) stiffness at the implanted level for all test conditions under each loading mode. Neutral zone as described by Panjabi (1992) is a measure of the angular deformation between the loading and unloading range of motion curves.^17^ This composite measure contains a proportional measure of hysteresis or energy loss during the range of motion cycle. This loss of energy, or increase in neutral zone, can be caused or affected by a multitude of factors including: viscoelastic relaxation of the soft tissues and TDA materials, a change in segmental stiffness, change in ROM, shift in the axis of rotation and the rate of loading. While segmental stiffness in the HFZ is a more reliable measure of quality of motion, NZ has been included for comparison to previous published studies.

A repeated‐measures anova with Bonferroni correction for multiple comparisons was used to assess the effects of the cervical disc prosthesis on the range of motion in each loading mode. Comparisons were made at C5‐C6 and C6‐C7 between the total disc replacement in the optimal position and the intact segment to determine if and to what extent the disc prosthesis restored normal spine function. Analysis was also performed between TDA optimal placement and both anterior and lateral placement at C5‐C6. The statistical data analyses were performed using the Systat 10.2 software package (Systat Software Inc., Richmond, California).

## 3 RESULTS

### 3.1 Range of motion

**Figure 3:**
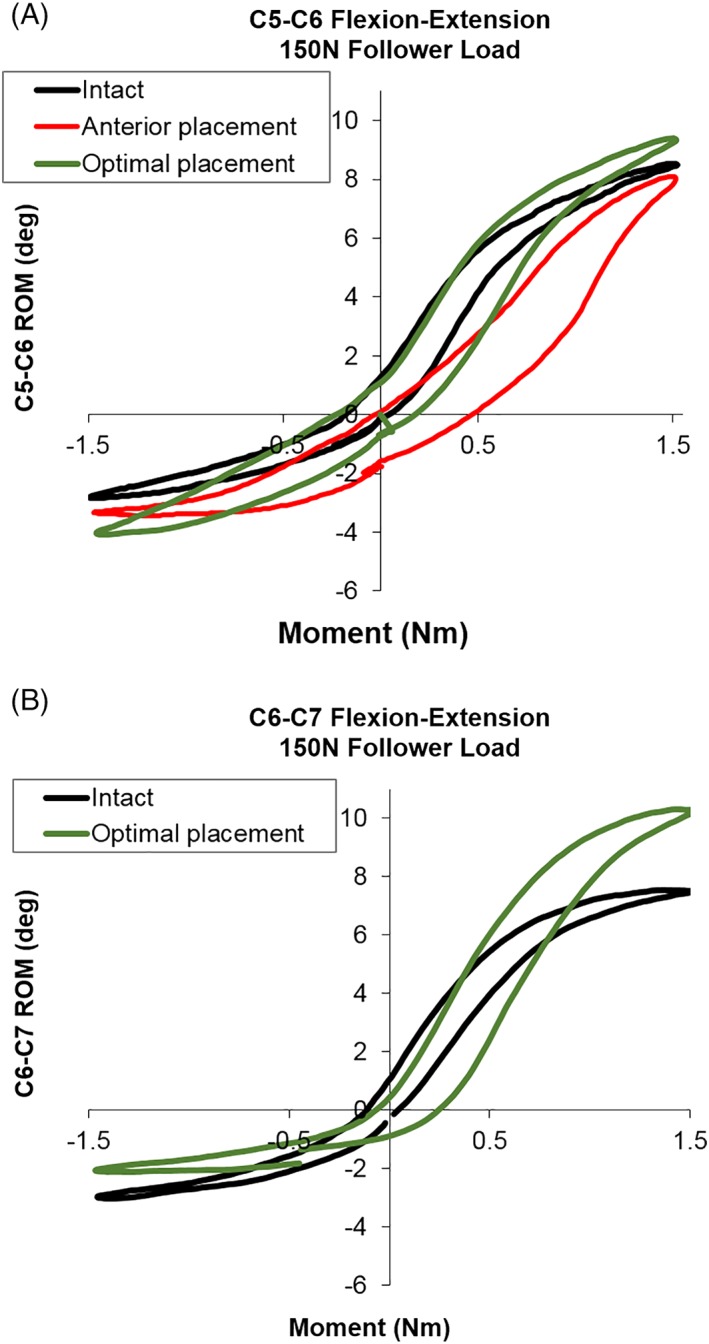
Representative segmental flexion‐extension load–displacement curves. (A) C5‐C6 ROM curves for intact, anterior and optimal TDA placement. (B) C6‐C7 ROM curves for intact and optimal TDA placement

**Figure 4:**
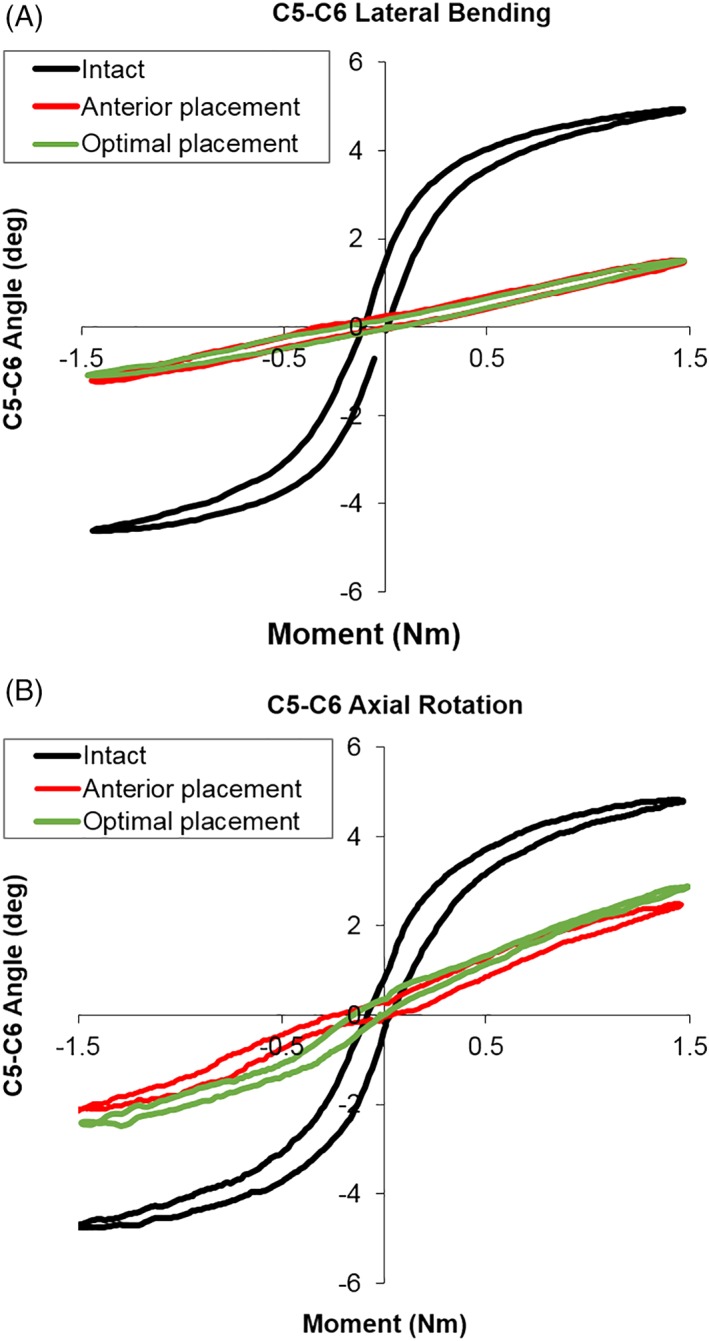
Segmental load–displacement curves at C5‐C6 under lateral bending (A) and axial rotation (B), for intact, anterior TDA placement, and optimal TDA placement

Representative applied moment vs angular displacement graphs (Figures 3 and 4) depict the classic sigmoidal behavior of the C5‐C6 and C6‐C7 motion segments in the intact condition, and after anterior and optimal placements of the TDA.

**Figure 5:**
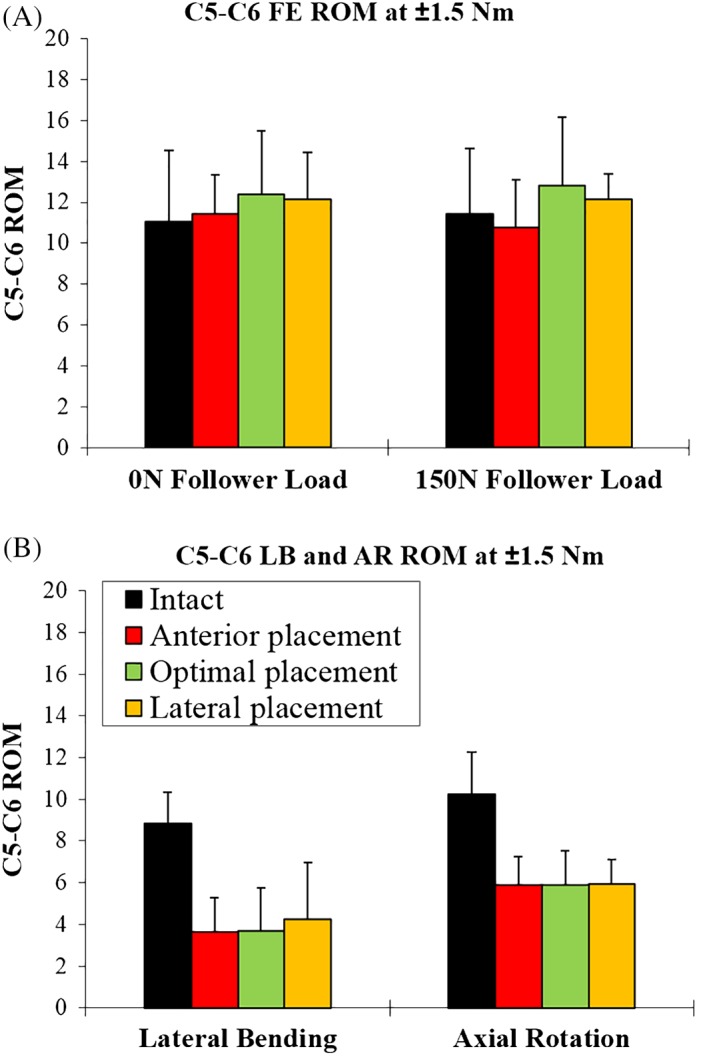
Segmental range of motion at C5‐C6 intact and after TDA placement in three locations. (A) Flexion‐extension ROM with and without follower load. (B) Lateral bending and axial rotation ROM

In the absence of a compressive preload (0 N), the C5‐C6 angular range of motion of the intact spine was 11.0 ± 3.5° in FE and increased slightly to 11.4 ± 1.9° after anterior TDA placement. Following optimal placement of the TDA, C5‐C6 ROM further increased to 12.4 ± 3.1°, but was not significantly different from either anterior placement or the intact condition (*P* > 0.05). Lateral placement of the cervical disc resulted in a slight decrease in C5‐C6 ROM (12.1 ± 2.3) compared to optimal placement (*P* > 0.05) (Figure 5A; Tables 2 and 4).

**Table 2:**
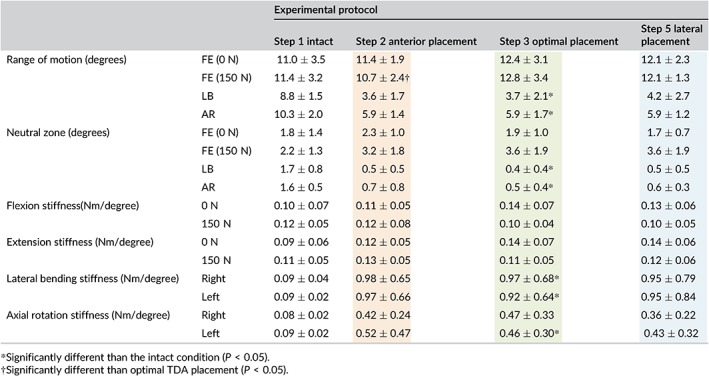
C5‐C6 segmental kinematics for each protocol step including: ROM, neutral zone, flexion stiffness and extension stiffness

Under a compressive load of 150 N, the intact C5‐C6 motion segment had a total angular motion of 11.4 ± 3.2° which decreased to 10.7 ± 2.4° following anterior placement of the TDA. Optimal placement of the TDA resulted in increased C5‐C6 ROM compared to anterior placement, from 10.7 ± 2.4° to 12.8 ± 3.4° (*P* = 0.014) but was not different than intact (*P* > 0.05). Lateral placement of the cervical disc did not significantly change the motion at C5‐C6 under compressive preload (*P* > 0.05) (Figure 5A; Tables 2 and 4).

**Figure 6:**
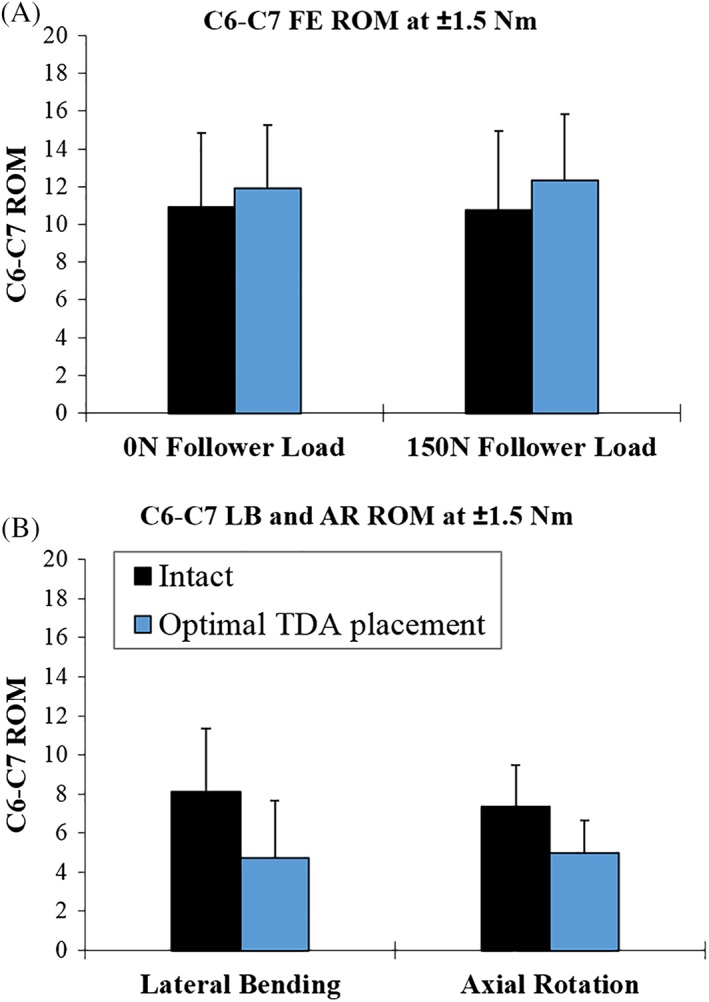
Segmental range of motion at C6‐C7 intact and with TDA placed in the optimal location. (A) Flexion‐extension ROM with and without follower load. (B) Lateral bending and axial rotation ROM

In the absence of a compressive preload, the C6‐C7 angular motion of the intact spine was 10.9 ± 3.9° in FE and increased to 11.9 ± 3.3° after optimal placement of TDA C6‐C7 (*P* > 0.05) (Figure 6A; Tables 3 and 4). Under a compressive load of 150 N, the intact spine had a C6‐C7 FE angular motion of 10.8 ± 4.2°. Optimal placement of the TDA caused an increase in C6‐C7 ROM to 12.3 ± 3.5° (*P* < 0.05) (Figure 6A; Tables 3 and 4).

**Table 3:**
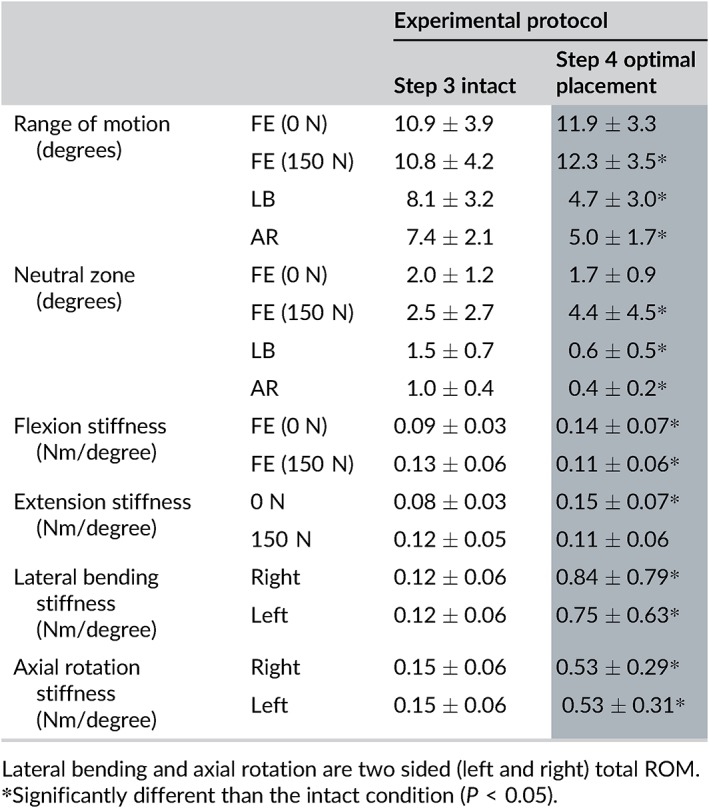
C6‐C7 segmental kinematics from each protocol step including: ROM, neutral zone, flexion stiffness and extension stiffness

**Table 4:** Statistical analysis of intact vs TDA placements (Bonferroni corrected *P*‐values)

		C5‐C6			C6‐C7
		Intact vs optimal	Anterior vs optimal	Lateral vs optimal	Intact vs optimal
ROM	FE (0 N)	1.000	0.406	1.000	0.198
	FE (150 N)	1.000	**0.014**	1.000	**0.045**
	LB	**0.001**	1.000	0.483	**0.000**
	AR	**0.000**	1.000	1.000	**0.001**
Neutral zone	FE (0 N)	0.894	0.081	0.577	0**.**278
	FE (150 N)	0.164	0.384	0.995	**0.035**
	LB	**0.022**	0.694	1.000	**0.001**
	AR	**0.000**	0.816	0.555	**0.001**
Flexion stiffness	0 N	1.000	0.440	1.000	**0.011**
	150 N	1.000	1.000	1.000	**0.044**
Extension stiffness	0 N	0.783	0.076	1.000	**0.005**
	150 N	1.000	1.000	1.000	0.233
Lat bend stiffness	Right	**0.036**	1.000	1.000	**0.044**
	Left	**0.036**	1.000	1.000	**0.031**
Axial rot stiffness	Right	0.056	1.000	0.223	**0.009**
	Left	**0.047**	1.000	0.565	**0.014**

Statistical significance is shown by bold *P*‐values with *P* ≤ 0.05.

Under moments of ±1.5 Nm in LB, the angular motion at C5‐C6 was significantly reduced from 8.8 ± 1.5° in intact to 3.7 ± 2.1° after optimal placement of the TDA (*P* < 0.01). Anterior and lateral placement of the TDA did not significantly change the C5‐C6 lateral bending ROM compared to optimal placement (Figure 5B; Tables 2 and 4).

C6‐C7 lateral bending ROM was also significantly reduced after TDA from 8.1 ± 3.2 ° intact to 4.7 ± 3.0° after optimal placement of the TDA (*P* < 0.01) (Figure 6B; Tables 3 and 4).

C5‐C6 AR, ROM was 10.0 ± 4.3° in the intact spine, which decreased to 5.9 ± 1.7° after optimal placement of the TDA (*P* < 0.01) (Figure 5B; Tables 2 and 4). Anterior and lateral placement were not significantly different than optimal placement (*P* > 0.05).

C6‐C7, intact AR motion was 7.4 ± 2.1°, which decreased to 5.0 ± 1.7° after optimal placement of the TDA (*P* < 0.01) (Figure 6B; Tables 3 and 4).

### 3.2 Flexion‐extension neutral zone

Regardless of the preload applied, C5‐C6 FE neutral zone was not affected by TDA placement in the anterior, optimal or lateral locations (*P* > 0.05) (Tables 2 and 4). Under 0 N preload, the mean change in NZ was less than 0.5° while under 150 N preload the mean change in NZ was less than 1.5°.

The C6‐C7 NZ was significantly affected by optimal placement of the TDA at C6‐C7 only under preload, increasing from 2.5 ± 2.7° to 4.4 ± 4.5° (*P* < 0.05) **(**Tables 3 and 4 **)**.

### 3.3 Stiffness

Placement (anterior, optimal, lateral) of the TDA at C5‐C6 did not significantly affect the flexion or extension stiffness either with or without preload (*P* > 0.05) compared to the intact condition. At C6‐C7 under 0 N compressive load, flexion and extension stiffness both increased significantly with optimal placement of the cervical disc (*P* < 0.05). However, under 150 N of compressive preload the flexion and extension stiffness both showed a decrease in segmental stiffness reaching significance in flexion (*P* < 0.05) **(**Tables 2 and 3 **)**.

### 3.4 Axis of rotation

**Figure 7:**
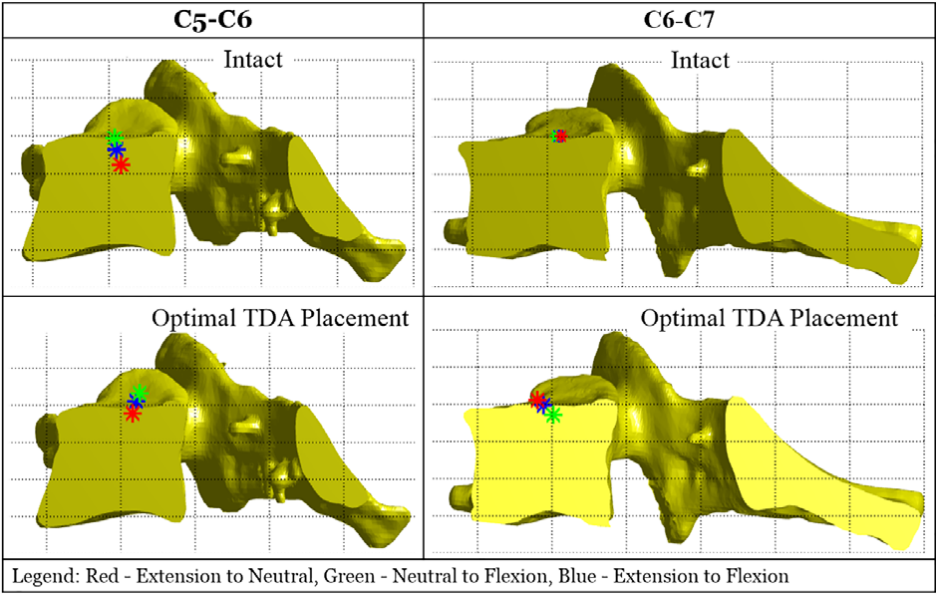
Specimen#6, C5‐C6 and C6‐C7 sagittal instantaneous axis of rotation shown on the segmental inferior vertebrae

Axis of Rotation was measured in two specimens (Specimen #5 and Specimen #6) using the specimen specific CT‐based kinematic analysis.^18^ The local anatomic coordinate system was located at the center of the superior endplate of the inferior vertebra of each motion segment (Anterior = +X, Cranial = +Z). The flexion‐extension axes of rotation of the two specimens are shown in Figure 7 and Table 5.

**Table 5:** Location of the C5‐C6 and C6‐C7 extension‐to‐flexion Axis of rotation projected on the sagittal plane

		Specimen 5		Specimen 6	
		X	Z	X	Z
C5‐C6	Intact	−3.4	−3.3	−0.9	−2.2
	TDA	−2.9	−3.1	−2.2	0.6
C6‐C7	Intact	−0.3	−1.5	2.5	−0.1
	TDA	−2.3	−0.9	1.9	0.0

The location of axis of rotation on the sagittal plane is in the local coordinate system of the inferior vertebra of each segment and is presented in mm. (Anterior = +X, Cranial = +Z).

## 4 DISCUSSION

The current study confirms that implantation of a cervical TDA prosthesis can provide near physiologic mobility at the implanted levels in flexion‐extension when compared to the native motion segments. Both quantity and quality of motion of the implanted motion segments closely approximated the healthy intact condition. Placement of a TDA in the laboratory during ex vivo testing can be achieved and documented with a high degree of accuracy due to the relaxed limitations on fluoroscopy and surgical time. Clinically, ideal TDA placement can be challenging due to limited direct visibility and difficulty in interpreting x‐rays in the AP plane with the retractors in place. Non‐optimal TDA placement was evaluated in this study by implanting the TDA anterior to the disc midline by 2 mm in the sagittal plane and lateral by 2 mm in the coronal plane in order to evaluate TDA performance in possible clinical scenarios. Placement variability did not negatively affect the TDA stiffness or neutral zone compared to optimal placement. ROM was slightly reduced with anterior placement under compressive preload, but the total ROM was still well within normative values.^19^

This study evaluated the kinematic response of the elastic‐core cervical disc arthroplasty using load control (flexibility) protocol.^20^ Moments of ±1.5 Nm were utilized based on laboratory experience and published literature.^11^, ^13^, ^14^, ^15^, ^16^ Kinematic response was evaluated with and without a compressive follower preload of 150 N. The follower preload is representative of the compressive forces induced by the stabilizing musculature and the mass of the head. A total of 150 N is within the range of forces that the cervical spine experiences in vivo.^21^ The follower preload was applied using optimized bilateral cables as described by Patwardhan et al.^11^, ^12^ While kinematic testing without preload represents a non‐physiologic condition, this study included testing without preload in order to compare to previous studies in which TDA testing was performed without compressive preload. In addition, testing without preload was performed to determine the sensitivity of this TDA to compressive preloads due to its non‐articulating design.

Normative in vivo and kinematic data from literature shows average C5‐C6 flexion‐extension ROM is between 12.5 ± 4.8 and 16.5 ± 5.0° and C6‐C7 ROM is between 12.5 ± 4.8 to 13.7 ± 5.1°.^19^, ^22^, ^23^ In lateral bending one sided in vivo motion is 4.3 ± 1.4 and 5.7 ± 1.9° for C5‐C6 and C6‐C7^24^ and average one sided motion in axial rotation is 5.4 ± 4.3 and 6.4 ± 2.5° for C5‐C6 and C6‐C7.^25^

While the ROM values in this ex vivo data set tend to be smaller than the above mentioned in vivo norms, caution must be used when making direct comparisons between these data sets. ex vivo data sets are collected in such a way that each data set (intact, anterior TDA placement, optimal TDA placement, etc.) is comparable to the previous and subsequent sets using the same loading rates and magnitudes. In vivo ROM experiments are subjective with each individual eliciting different levels of muscle recruitment which cause varying segmental moments and compressive loads. Despite these seemingly desperate methodologies, the intact ROM values of the specimens in this sample fall within the normative in vivo ranges of motion in all three testing directions. Optimal placement of the elastic‐core TDA resulted in a small (1.4°) non‐significant increase in flexion‐extension ROM at C5‐C6 with and without compressive preload. At C6‐C7 there was a modest (1.5°) but statistically significant increase in ROM with TDA under 150 N follower load.

Lateral bending and axial rotation ROM both decreased after TDA implantation. This phenomenon is well documented in the literature. Single spherical bearing, ball and socket TDA designs were investigated by Puttlitz et al (ProDisc‐C) and Snyder et al (Discover Cervical TDA).^4^, ^14^ In lateral bending the authors noted decreases in ROM of 37% and 50% respectively after placement of these fixed center of rotation designs. In axial rotation the changes in ROM were less dramatic, limiting motion by 27% and 22%.^4^ The most recent evaluation of the M6 six‐degree‐of‐freedom prosthesis showed similar results with a decrease in lateral bending of 44% and axial rotation of 16%.^11^ These aforementioned studies were performed in load control mode to 1.5 Nm. Other studies have been published that show larger amounts of lateral bending and axial rotation after TDA.^2^, ^26^, ^27^ However, these studies were run in hybrid control, in which specimens were run to the same angular endpoints before and after arthroplasty, possibly resulting in loads in excess of 1.5 Nm. For this reason the results of these studies cannot be compared to the present study or other load control studies from the literature.

During TDA implantation, the anterior annulus was resected leaving a window just wide enough for implant insertion. Though not a requirement for implantation of this TDA, the posterior longitudinal ligament (PLL) was resected while leaving the anterolateral and posterolateral annulus as well as uncinates intact. The decrease in lateral bending and axial rotation seen in this study can likely be attributed to several factors including a change in the segmental axis of rotation. This change in axis of rotation can be attributed to the device kinematics as well as a change in the motion segment height and segmental lordosis, and the tension of retained soft tissues. The RHINE disc has an elastic polymer core, and as such has some ability to accommodate a changing axis of rotation as the motion segment moves through different planes of motion. This accommodation is limited in the immediate post operative time frame by the increased tissue tension necessary to provide appropriate segmental height restoration and ligamentotaxis to prevent device migration. Future clinical studies can provide an improved understanding of preserved lateral bending motion after TDA and if lateral bending ROM increases with successive follow‐up evaluations.

Quality of segmental motion is the evaluation of how motion between the flexion and extension endpoints occurs and can be evaluated using motion segment stiffness in the high flexibility zone (HFZ).^28^ The HFZ is the region around neutral posture in which the segmental ligamentous structures remain untensioned. This is the region in which the vast majority of activities of daily living occur. In the cervical spine, the motion segment stiffness in the HFZ is governed by the disc and facet interaction as well as muscle activation patterns and forces. Panjabi suggested that decreased segmental stiffness around the neutral posture would place increased demand on the spinal musculature to stabilize the motion segment.^17^ This increased muscle activity will increase segmental stresses leading to pain. Panjabi used a measure of neutral posture laxity termed the neutral zone (NZ). This measure was popularized before the era of continuous cyclic loading of spine specimens. Measure of the NZ was simply the angular difference in degrees between the loading curve in flexion and the loading curve in extension. The NZ measure is a function of the viscoelastic relaxation that has taken place during the testing and is a function of motion segment stiffness, segmental range of motion, rate of loading and soft tissue integrity or health. Under the assumption that ROM and loading rate have not changed, NZ can provide a simple measure of segmental instability. When using NZ to evaluate segmental kinematics, care must be taken to evaluate the NZ not at 0 N of loading, but rather at the center of the high flexibility zone.^28^ This is important due to the fact that the true neutral posture for cadaveric specimens is not known and not all motion segments reach their neutral posture simultaneously or necessarily at zero moment.

The results of this study of the RHINE cervical disc arthroplasty show that segmental stiffness in flexion and extension tend to increase in the absence of follower preload and decrease slightly with 150 N of compressive follower preload. This can primarily be observed at C6‐C7 and may be due to the compressive preload causing slack in the ligamentous tissues allowing a slight decrease in resistance to motion. With the decrease in lateral bending and axial rotation ROM, the segmental stiffness in these directions similarly increased beyond the intact condition.

### 4.1 ROM of anterior placement

Optimal placement of the RHINE cervical disc arthroplasty resulted in no significant change in flexion‐extension ROM compared to anterior placement under in the absence of compressive follower load (0 N). With the addition of 150 N preload, flexion‐extension ROM increased significantly in the optimal placement compared to anterior placement.

### 4.2 ROM of lateral placement

Placement of the TDA 2 mm lateral to the disc midline had no significant effect on flexion‐extension (0 N and 150 N), lateral bending or axial rotation ROM compared to optimal placement.

### 4.3 Limitations

Interpretation of these results requires some consideration of the study limitations. Biomechanical testing at best mimics the immediate postoperative condition, and therefore changes in the soft tissues, such as annular scar tissue formation, and bony remodeling, are not incorporated, although anular relaxation may be largely accounted for.^29^ A second noteworthy limitation of this study is the inability to entirely replicate in‐vivo physiologic loading. Although application of the follower load technique provides a key component of the in vivo environment,^12^ the complicated musculature of the neck creates loading conditions nearly impossible to reproduce completely on a cadaveric spine in the laboratory environment.

Finally, this research study was performed on relatively healthy specimens with no significant disc degeneration in order to eliminate confounding factors such as bridging osteophytes, facet and advanced disc degeneration, etc. A major advantage of testing on relatively healthy specimens is the ability to compare postoperative motions to the native disc. This provides a specimen‐specific and motion segment specific control for data comparison.

## 5 CONCLUSIONS

This six degree of freedom elastic‐core disc arthroplasty device effectively restored flexion‐extension motion to intact levels at both C5‐C6 and C6‐C7. In LB the TDA maintained 42% ROM at C5‐C6 and 60% at C6‐C7. In AR 57% of the ROM was maintained at C5‐C6 and 70% at C6‐C7. These findings are supported by literature which shows cervical TDA results in restoration of approximately 50% ROM in LB and AR, which is a multifactorial phenomenon encompassing TDA design parameters and anatomical constraints. Anterior placement of this elastic‐core TDA device shows motion restoration similar to optimal placement suggesting its design may be less sensitive to sub‐optimal placement than mechanical TDAs with moving parts. In this two level study, the data suggests that this device restores ROM to preoperative levels in flexion‐extension. This new generation of TDA offers an alternative to fixed axis of rotation and articulating devices.

## CONFLICTS OF INTEREST

R.D.G., MD: Royalties: Alphatec; Stock Ownership: Spinal Motion; Private Investments: Spinal Ventures; Consulting: DePuy Synthes; Speaking and/or teaching arrangements: Synthes; Scientific Advisory Board: K2M, Spinal Kinetics (Stock options), Nanovis, Crocker Technologies, MiMedx; Fellowship Support: OREF (Amount not disclosed, Paid directly to institution/employer), Medtronic Neurological Division (Paid directly to institution/employer). L.I.V., MD, PhD: Nothing to disclose. R.M.H., MS: Nothing to disclose. S.K., PhD: Nothing to disclose. G.C., MS: Nothing to disclose. K.R.B., MS, MHA: Nothing to disclose. S.W., BS: Nothing to disclose. J.R., BS: Employee of K2M Inc. N.P., BS: Employee of K2M Inc. A.G.P.: Funding support from K2M (Paid directly to Institution).
